# ERK/pERK expression and B-raf mutations in colon adenocarcinomas: correlation with clinicopathological characteristics

**DOI:** 10.1186/1477-7819-10-47

**Published:** 2012-02-29

**Authors:** Georgia Levidou, Angelica A Saetta, Fanie Gigelou, Maria Karlou, Polyanthi Papanastasiou, Angeliki Stamatelli, Nikolaos Kavantzas, Nikolaos V Michalopoulos, George Agrogiannis, Efstratios Patsouris, Penelope Korkolopoulou

**Affiliations:** 1 Department of Pathology, National and Kapodistrian University of Athens, Medical School, 75 Mikras Asias street, Greece 11527

## Abstract

**Background:**

Colorectal (CRC) carcinogenesis through various morphological stages has been linked to several genetic and epigenetic changes. The Raf/MEK/ERK (MAPK) signal transduction cascade is an important mediator of a number of cellular fates.

**Methods:**

In this study, we investigated the presence of B-raf and K-ras mutations in 94 consecutive cases of primary colon adenocarcinoma in correlation with the immunohistochemical expression of total and activated ERK and the expression of mismatch repair proteins (MMR) hMLH1 and hMSH2 as well as their correlations with standard clinicopathological parameters.

**Results:**

The immunostaining pattern for total and activated ERK was nuclear and cytoplasmic. hMLH1 and hMSH2 proteins were preserved in 45/63 (71.43%) cases and 35/53 (66.04%) cases respectively. Total ERK nuclear expression, was positively correlated with tumor stage (p = 0.049), whereas nuclear pERK expression was positively correlated with histological grade (p = 0.0113) and tumor stage (p = 0.0952), although the latter relationship was of marginal significance. DNA sequencing showed that 12 samples (12.7%) had a mutation in B-RAF Exon 15 and none in Exon 11, whereas 22 (23.4%) had a K-ras mutation. Disruption of the MAP kinase pathway-either through K-ras or B-raf mutation-was detected in 37% of all the examined cases, although the overexpression of total and activated ERK1/2 was not correlated with the mutational status of K-ras or B-raf genes. Finally, the preservation of hMLH1 or hMSH2 immunoexpression was not correlated with the presence of B-raf and/or K-ras mutations.

**Conclusions:**

In this study, we present evidence that ERK activation occurs in a K-ras or B-raf -independent manner in the majority of primary colon cancer cases. Moreover, B-raf mutations are not associated with mismatch-repair deficiency through loss of hMLH1 or hMSH2 expression. Activated ERK could possibly be implicated in tumor invasiveness as well as in the acquisition of a more aggressive phenotype.

## Background

Colon cancer is the fourth most common malignancy in terms of both incidence and mortality worldwide [1]. The development of colon cancer is a complex multistep process dependent on both genetic and environmental factors, in which oncogenes, tumor suppressor genes as well as genes involved in DNA damage recognition and repair have been implicated. Gaining insight into the molecular pathways involved in the progression of colon cancer is imperative for the development of innovative individualised cancer treatment strategies.

Colorectal (CRC) carcinogenesis through various morphological stages has been linked to several genetic and epigenetic changes. Until recently, two main pathways of sporadic colorectal carcinogenesis were proposed: a) the chromosomal instability (CIN) pathway which affects proto-oncogenes and tumor suppressor genes and is characterized by alterations of chromosomal number and structure, and b) the microsatellite instability (MSI) pathway, responsible also for Lynch syndrome CRCs, which features size variations of repeated nucleotides (microsatellites) mainly in non coding sequences, due to defects in the mismatch repair system (hMLH1, hMSH2, mHMS6, PMS1, PMS2) [2,3]. In addition to CIN and MSI pathways, a third pathway, the epigenetic instability, which is thought to be largely driven by hypermethylation-induced silencing of tumor suppressor-like genes, has been implicated in the progression of colorectal carcinogenesis [4]. According to this notion, contemporary literature suggests that CRC in general develops through two independent pathways that involve sequences of genetic and epigenetic alterations associated with pathological and clinical features: the adenoma pathway in 70-80% and the newly recognized, the serrated pathway in the remaining 20-30% [5]. The somatic molecular features which characterize the newly introduced serrated pathway to CRC include activating mutations in *B-raf *[6] and widespread hypermethylation of gene promoters (CIMP) [7] with or without MSI [6].

The kinases of mitogen-activated protein (MAP) kinase superfamily participate in signaling cascades conserved through evolution, which transduce extracellular signals into intracellular responses. MAP kinases are major components of pathways controlling embryogenesis, cell differentiation, proliferation and death. The Ras/Raf/mitogen-extracellular signal-regulated kinase 1/2 (MEK1/2)/extracellular signal regulated kinase 1/2 (ERK1/2) cascade is activated by mitogenic factors, differentiation stimuli and cytokines [8,9]. The Raf family of protein kinases, which is one class of Ras effectors, phosphorylates the dual specific MAP kinases MEK1 and MEK2, which in turn phosphorylate and activate the effector MAP kinases ERK1 and ERK2 [9]. ERKs are multifunctional serine/threonine kinases that target a vast array of substrates localized in all cellular compartments, such as protein kinases, signaling effectors, receptors, cytoskeletal and nuclear proteins and transcription factors, that can influence cell fate [9-11].

Importantly, MAP kinases are capable of affecting gene expression via intermediary kinases by phosphorylating proteins in the cytoplasm, but also translocate to the nucleus, a critical step for the fulfilment of many cellular functions of ERK, such as gene transcription, cell proliferation and differentiation [12]. Through phosphorylation of these various substrates, constitutively activated ERKs are able to influence many of the hallmarks of carcinogenesis, as defined by Hanahan and Weinberg [13]. Constitutive activation of this pathway has been observed in several human malignancies and cell lines such as breast, colon, thyroid carcinomas and melanomas and provides a potent promitogenic force resulting in uncontrolled proliferation and differentiation [14,15].

The current study investigates the presence of mutations in K-ras and B-raf genes in colorectal carcinoma, in correlation with MAP kinase ERK expression and the expression of mismatch repair proteins (MMR) hMLH1 and hMSH2, attempting to elucidate the involvement of these MAP kinases in the development of colorectal cancer, as well as their correlations with standard clinicopathological parameters.

## Methods

### Patients

This is a retrospective study of 94 consecutive cases of primary colon adenocarcinoma (53 men and 41 women, median age of 60 years -range 35 to 82) for whom archival material from primary tumor resection surgical specimens was available. None of the patients had received chemotherapy or radiation before surgery. All cases were reviewed by two experienced pathologists (PK, GL) and assigned a histological grade according the standards laid down in the latest WHO classification of colon carcinoma [16]. There were 10 grade 1, 64 grade 2 and 20 grade 3 carcinomas. According to the latest TNM system of cancer staging adopted by the American Joint Committee on Cancer and the International Union Against Cancer (AJCC/IUCC), tumors were classified as stage I: 7 cases (T1N0M0, T2N0M0), stage II: 36 cases [stage IIA (T3N0M0), IIB (T4aN0M0) IIC (T4cN0M0), stage III: 47 cases [stage IIIA (T1-T2N1M0, T1N2aM0), IIIB (T3-T4aN1M0, T2-T3N2aM0, T1-T2N2bM0) and IIIC (T4aN2aM0, T3-T4aN2bM0, T4bN1-2M0)] and stage IV:4 (Any T Any N M1) cases.

### Immunohistochemical analysis

Immunohistochemical analysis was performed in the same blocks that were used for molecular analysis. Accordingly, the tumor tissue that was available for immunohistochemistry was exhausted in some cases. Thus, the immunohistochemical expression of ERK protein was available in a subset of 55 specimens, the expression of pERK protein in 45 specimens, the expression of hMLH1 in 63 cases and the expression of hMSH2 in 53 cases. However, the distribution of mutations in K-ras and B-raf (exon 15 or 11) did not differ among the specimens for which immunohistochemical analysis was available and those that immunohistochemistry was not available, a fact that suggests that there was not any significant bias in this regard. ERK and pERK immunostaining was performed using a rabbit polyclonal (#9488, p44/42 MAP kinase, Cell Signaling, Beverly, MA) for ERK1/2 and a mouse monoclonal antibody (sc-7383, (E-4) Santa Cruz Biotechnology, Inc., California, USA) for pERK, whereas hMLH1 and hMSH2 immunostaining using mouse monoclonal antibodies (#554073, G168-15 for hMLH1, Pharmingen, San Diego, California and clone GB-12, for hMSH2, Oncogene Cambridge, MA). Sections (3 to 4 μm) were deparaffinized, rehydrated, immersed in 3% H_2_O_2 _for 30 min and microwaved at 750 W in 0.01 M citrate buffer (pH 6.0) for 15 min and left to cool down in TBS. Sections were incubated with ERK. pERK, hMLH1 and hMSH2 antibodies overnight at room temperature (37°C), at a dilution 1:100, 1:350, 1:180 and 1:180 respectively. Immunostaining was performed using the standard avidin-biotin complex (Ultra Vision Polymer, LabVision) and visualized with diaminobenzidine tetrahydrochloride solution.

Light microscopic evaluation of immunostained slides was performed independently by three (PK, NK, GL) experienced pathologists without previous knowledge of the clinical information. If a discrepancy occurred between the three assessments, the slides were reassessed jointly. In each case 1000-1500 neoplastic cells throughout the section were counted at high-power magnification. Nuclear and cytoplasmic pERK and ERK expression were evaluated separately. Nuclear or cytoplasmic pERK and ERK labeling index was defined as the percentage of neoplastic cells with nuclear or cytoplasmic immunoreactivity out of the total number of neoplastic cells counted. According to the percentage of positively stained neoplastic nuclei, each case was considered to display reduced (< 20%) or preserved (≥20%) expression of hMSH2 and hMLH. This is an arbitrary cut-off chosen for the purposes of this study, since no uniform threshold has been established in the respective literature. In this context studies evaluating the expression of MMR proteins in colorectal cancer have used cut-offs ranging from 5-50%, whereas in other tumors the more frequently cut-off value used is 20% [17-21].

### Genomic DNA isolation

15 μm sections were used for DNA extraction. The samples were digested overnight at 55°C using 200 μl of digestion buffer consisting of 50 mM Tris, 1 mM EDTA, 0.5% SDS and 200 μg/ml Proteinase K. Genomic DNA was extracted with phenol-chloroform and precipitated in ice-cold ethanol. The DNA was redissolved in distilled water and quantified by spectrophotometry at 260 nm under UV light.

### PCR

200 ng of total DNA were amplified in a 50 μl reaction mixture containing 25 pmoles of each primer, 25 mM each dNTP, 1.5 mM MgCl_2_, 1 mM KCl, 0.1% gelatin and 1.5 U Taq DNA polymerase (Advantage, Clontech, Takara, USA). The profile used in the Progene Techne thermal cycler was: 5 min at 95°C once; 30 sec at 95°C, 40 sec at 52-56°C, 1 min at 72°C for 40 cycles; 7 min at 72°C once. Sequences of the primers used are the following:

K-ras: F -actgaatataaacttgtggtagttggacct-

K-ras: R -tcaaagaatggtcctggacc-

B-raf exon 15: F- TCA TAA TGC TTG CTC TGA TAG GA-

B-raf exon 15: R- GGC CAA AAA TTT AAT CAG TGG A-

### RFLP analysis

5-10 μl aliquots of the PCR product were digested with BstNI to reveal the presence of mutations in codon 12 of *K-ras *gene. Incubation was performed at 60°C for 3 hrs. The digestion products were then visualized by ethidium bromide staining under UV light after electrophoresis on a 4% (3:1 Nusieve agarose) gel (Lonza, Cologne, GmbH). The size of the PCR product for K-ras amplification is 157 bp and includes two restriction sites for BstNI restriction endonuclease. Thus the normal K-ras allele is indicated by the presence of a 114 bp band in the gel whereas the mutant K-ras allele by a 143 bp band. Heterozygous mutant cases display both bands of 143 bp and 114 bp

### SSCP analysis

PCR products were screened for mutations in exons 11 and 15 of the B-raf gene. Firstly, PCR products were diluted in 10 μl formamide-dye solution (95% formamide, 20 mM EDTA, 0.05% bromophenol blue, 0.05% xylene cyanol), denatured for 7 min at 95°C and kept on ice until loaded onto a 0,5× MDE gel (BMA, USA). Electrophoresis was carried out at 3 W for 16-18 hours at 4°C. After electrophoresis, the gel was silver stained and examined for abnormal band patterns. The analysis of all cases displaying abnormal band patterns was repeated at least twice.

### Sequencing

For *B-raf *mutation analysis the Big Dye terminator cycle sequencing kit was used. The aberrant SSCP cases were sequenced at least twice on an ABI prism 310 Genetic analyzer (Perkin-Elmer, California, USA). PCR primers were used for sequencing as well.

### Statistical analysis

In the basic statistical analysis pERK and ERK nuclear or cytoplasmic expressions were treated as continuous variables, whereas hMLH1 and hMSH2 expression levels were treated as categorical variables. The relationships among ERK, pERK, hMLH1, hMSH2 expression and clinicopathological parameters, such as grade and stage were tested with non parametric tests (Spearman correlation coefficient, Kruskal-Wallis ANOVA and Mann Whitney U test), as appropriate. The respective correlations among mutation status (regarding K-ras or B-raf) and clinicopathological or immunohistochemical data were calculated using Fisher's exact test or Mann-Whitney U test. Statistical calculations were performed using the Statistical package STATA 9.0 for Windows. All results with a two-sided p ≤ 0.05 were considered statistically significant.

## Results

### Expression of ERK, pERK, hMLH1 and hMSH2 in colorectal carcinomas (Figure 1)

**Figure 1:**
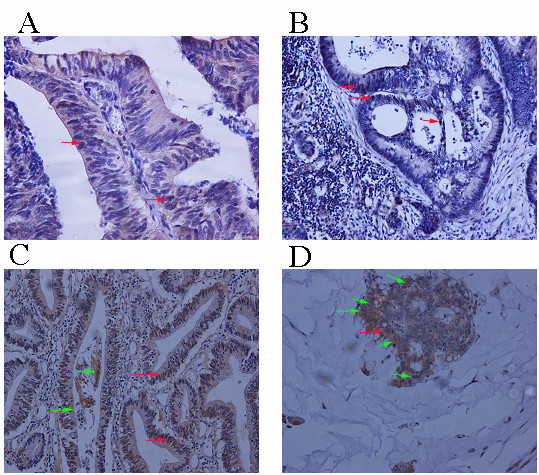
**A. Grade 1, stage I colorectal carcinoma, displaying minimal nuclear pERK staining**. B. Grade 3, stage III colorectal carcinoma, displaying increased nuclear pERK staining. C, D. ERK nuclear and cytoplasmic immunoreactivity in grade 1, stage II (C) and grade 3, stage III with production of extra-cellular mucin (D).

The detailed results of immunohistochemical analysis are shown in Additional file 1. The immunostaining pattern for ERK was nuclear and cytoplasmic. All cases (55/55) displayed either nuclear and/or cytoplasmic immunostaining. Nuclear immunoreactivity for ERK was detected in 85% (47/55) of cases, whereas cytoplasmic immunoreactivity in 92.7% of cases (51/55). In 43 cases (78.18%) concurrent cytoplasmic and nuclear positivity was recorded. pERK immunoreactivity was mainly nuclear found in 68.89% (31/45) of cases, while a small proportion of specimens displayed (11 cases, 24.4%) cytoplasmic positivity. In eight of these cases (17.78%) there was simultaneous nuclear and cytoplasmic pERK immunoexpression. Normal colorectal mucosa was present in 39 of the 55 examined slides and displayed membranous ERK immunoreactivity in the apical aspect of the superficial epithelium.

Nuclear immunoreactivity levels of ERK varied widely among tissue samples. We found that 13 samples had no expression (12.73%), 22 had a weak expression (40%, from 1 up to 15%), 13 had moderate expression (23.64%, from 15 up to 50%), whereas 13 had strong immunoexpression (23.64%, > 50%). Cytoplasmic immunoreactivity of ERK was in higher levels, since the majority of samples (40 in 55 cases, 72.73%) displayed strong positivity (> 50%). Most samples with strong cytoplasmic expression displayed increased nuclear expression, an observation which was confirmed with the calculation of the Spearman's correlation coefficient (rho = 0.3361, p = 0.0121).

pERK nuclear and cytoplasmic immunoexpression displayed a narrower range of variation, ranging from no expression up to 20% and 15% of tumor cells respectively. Also, there was no significant correlation between pERK nuclear and cytoplasmic expression (Spearman's correlation coefficient, p > 0.10). Normal colorectal mucosa showed minimal nuclear pERK expression whereas there was diffuse cytoplasmic pERK expression (results available in 36 cases in which there was normal mucosa adjacent to the tumor).

Additionally, there was no correlation between nuclear or cytoplasmic ERK labelling index and the presence of nuclear and/or cytoplasmic pERK immunoexpression (Mann Whitney U test, p > 0.10).

As far as hMLH1 and hMSH2 protein expression is concerned, it was preserved in 45/63 (71.43%) cases and 35/53 (66.04%) cases respectively. Adjacent normal colorectal mucosa preserved the expression of hMLH1 (available in 44 cases) and hMSH2 (available in 36 cases).

### Associations between ERK, pERK, hMLH1 and hMSH2 with clinicopathological features

A significant association emerged between tumor's stage and ERK nuclear expression levels, suggesting lower expression levels (< 15%) in advanced tumors (Fisher's exact test, p = 0.049, Table 1). However, ERK nuclear immunoexpression was not correlated with histological grade, tumor location (Kruskal Wallis ANOVA, p > 0.10, Table 1) patients' gender or age (Spearman, p > 0.10, Table 1). Moreover, cytoplasmic ERK immunoreactivity was not correlated with any of the examined clinicopathological parameters (p > 0.10, Table 1).

**Table 1:** Associations between nuclear ERK expression and clinicopathological parameters in 55 patients with colorectal carcinoma.

Variables		ERK nuclear			ERK cytoplasmic expression		
	**N**	**Low expression group (< 15%)**	**High expression group (≥15%)**	**p-value**	**Low expression group (< 50%)**	**High expression group (≥50%)**	**p-value**

**Gender**							

Male	33	16	17	0.99	7	26	0.235
			
Female	22	10	12		8	14	

***TNM stage***							

I	4	0	4	0.049	2	2	0.298
			
II-IV	51	26	25		13	38	

**Grade**							

I	23	20	25	> 0.10	10	33	0.404
			
II/III	8	4	4		3	5	

***Tumor location***							

Right colon	30	17	13		7	23	0.551
	
Left colon	25	9	16	0.99	8	17	

		**Median (range)**	**Median (range)**		**Median (range)**	**Median (range)**	

***Age (years)***	55	66.5 (51-77)	61 (35-83)	0.5562	59 (56-76)	66.5 (35-83)	0.5622

Results of Fisher's exact test.

**Figure 2:**
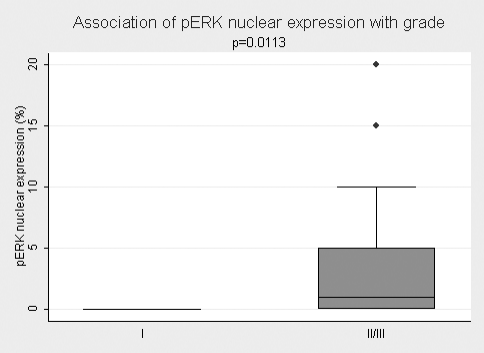
**Schematical representation of the association between nuclear pERK expression and histological grade**. Tumors with grade II/III tended to display higher pERK nuclear immunoreactivity compared to those with grade I (p = 0.0113, Mann- Whitney U test).

pERK nuclear immunoreactivity was positively correlated with tumor grade (I vs II/III, Mann Whitney U test, p = 0.0113, Table 2, Figure 2). Thus, grade (II and III) tended to display higher pERK nuclear immunoreactivity levels. Accordingly, pERK nuclear immunoreactivity was mainly reported in advanced stage tumors (Fisher's exact test, p = 0.0952, Table 2), although this relationship was of marginal significance. The relationships between nuclear pERK expression levels and tumor location or patients' age, as well as pERK cytoplasmic immunoreactivity and all clinicopathological features failed to attain statistical significance (p > 0.10, Table 2). On the contrary the preservation of hMLH1 and hMSH2 expression was not correlated with tumor's stage, histological grade, tumor location or patients' age and gender (p > 0.10, table 3).

**Table 2:** Associations between nuclear and cytoplasmic pERK expression and clinicopathological parameters in 45 patients with colorectal carcinoma.

		*pERK nuclear LI*			*pERK cytoplasmic LI*		
	***N***	***Median***	***Range ***	***P value***	***Median***	***Range ***	***P value***

***Gender***							

Male	24	1	0-15		0	0-10	

Female	21	1	0-20	0.6999	0	0-15	0.9518

***Histological grade***							

I	4	0	0-0		0	0-0	

II/III	41	1	0-20	0.0113	0	0-15	0.2504

***TNM stage***							

I	5	0	0-10		0	0-0	

II-IV	40		0-20	0.0952	0	0-15	0.1873

***Tumor location***							

Right colon	33	1	0-20		1	0-20	

Left colon	12	1	0-10	0.3941	5	0-10	0.4452

							

***Age (years)***	45	R = 0.3941, p = 0.5687			R = 0.3153, p = 0.1529		

Results of Mann Whitney U test.

**Table 3:** Associations between hMLH1 and hMSH2 expression and clinicopathological parameters in 53 and 63 patients with colorectal carcinoma respectively.

Variables	hMLH1 expression				hMSH2 expression			
	**N**	**Reduced (< 20%)**	**Preserved (≥20%)**	**p-value**	**N**	**Reduced (< 20%)**	**Preserved (≥20%)**	**p-value**

**Gender**								

Male	34	10	24	0.99	30	10	20	0.99
			
Female	29	8	21		23	8	15	

***TNM stage***								

I	5	1	4	0.99	6	3	3	0.397
			
II-IV	58	17	41		47	15	32	

**Grade**								

I	8	1	7	0.421	6	2	4	0.99
			
II/III	55	17	33		47	16	31	

***Tumor location***								

Right colon	36	11	25		33	11	22	0.99
	
Left colon	27	7	20	0.782	20	7	13	

		**Median (range)**	**Median (range)**			**Median (range)**	**Median (range)**	

***Age (years)***	63	58 (53-73)	63(40-83)	0.2558		60(45-83)	60(40-81)	0.9607

Results of Fischer's exact test.

### Associations between ERK, pERK, hMLH1 and hMSH2 expression levels with B-RAF and/or K-RAS mutational status

**Figure 3:**
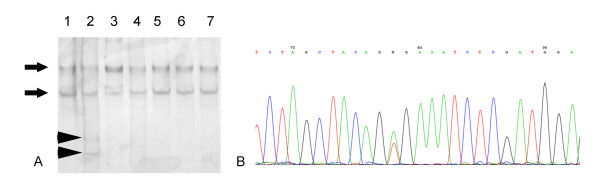
A. Representative SSCP* analysis of B-raf gene, exon 15 of seven colorectal cancer cells. Lanes 1, 3-7: tumor samples with normal band pattern (black arrows). Lane 2: tumor sample with the typical band pattern of a V600E mutation (arrow heads). *SSCP: Single strand conformation polymorphism. B. Sanger heterozygous analysis showing a heterozygous B-raf mutation (exon 15) at codon 600 (p.V600E, c.1799T > A).

**Figure 4:**
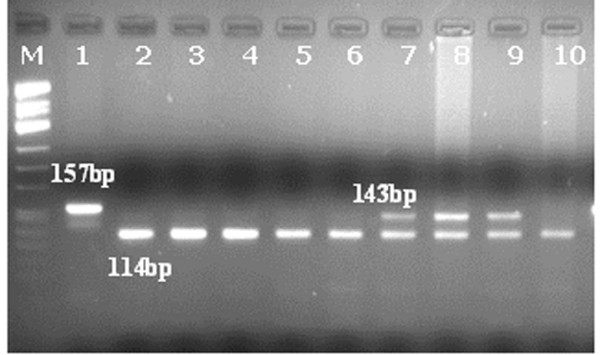
**Mutational analysis of K-ras codon 12 by PCR-RFLP (digestion with BSTNI restriction endonuclease)**. PCR product size: 157 bp, Normal K-ras allele: 114 bp, Mutant K-ras allele: 143 bp. Heterozygous mutant case: 143 bp and 114 bp. Μ = molecular weight marker pUC Mix 8. Lane 1 = Undigested PCR product (157 bp). Lanes 7-9 = Mutant samples (143 bp and 114 bp). Lanes 2-6 & 10 = Normal samples (114 bp).

The results of molecular analysis are shown in Additional file 1. Of the 94 samples, 12 had a mutation in B-raf Exon 15 (Figure 3a,b) and none in Exon 11, whereas 22 had a K-ras mutation (Figure 4). In the subset of 55 specimens that were available for immunohistochemical evaluation of ERK protein tissue expression 4 had a B-raf and 14 a K-ras mutation (Table 4). Most of the cases with nuclear and cytoplasmic ERK expression did not have a K-RAS mutation (79.09%, 34/43), although this observation was not statistically significant (Table 4). The same applied to the cases with the presence of either nuclear (77.08%, 37/48) or cytoplasmic (76%, 38/50) expression. Moreover, three of the 4 cases with B-raf mutation had positive nuclear and cytoplasmic ERK expression (75%), whereas the fourth displayed only nuclear immunoreactivity. Importantly, the presence of either B-raf or K-ras mutation was marginally associated with the presence of cytoplasmic ERK expression (Fisher's exact test, p = 0.097), suggesting that the proportion of cytoplasmic ERK positivity is marginally higher in the mutated cases when compared to the unmutated ones.

**Table 4:** Correlations of the presence of K-ras and B-raf mutations (exon 15) with immunohistochemical results in patients for whom staining data were available.

	K-ras Mutation			B-raf Exon 15 mutation		
	**Absent**	**Present**	**p**	**Absent**	**Present**	**p**

**ERK nuclear expression (n = 55)**						

Absent	4	3		7	0	

Present	37	11	0.354	44	4	0.99

**ERK cytoplasmic expression (n = 55)**						

Absent	3	2		4	1	

Present	38	12	0.592	47	3	0.325

**ERK nuclear and cytoplasmic expression (n = 55)**						

Absent	7	5		11	1	

Present	34	9	0.259	40	3	0.99

**pERK nuclear expression (n = 65)**						

Absent	11	3		13	1	

Present	24	7	0.99	27	4	0.99

**pERK cytoplasmic expression(n = 65)**						

Absent	27	7		29	5	

Present	8	3	0.687	11	0	0.313

**pERK nuclear and cytoplasmic expression (n = 65)**						

Absent	29	8		32	5	

Present	6	2	0.99	8	0	0.357

**hMLH1 expression (n = 63)**						

Reduced	13	5		15	3	

Preserved	33	12	0.99	41	4	0.397

**hMSH2 expression (n = 53)**						

Reduced	12	6		13	5	

Preserved	24	11	0.99	32	3	0.104

Results of Fischer's exact test.

In the subset of 45 specimens that were available for pERK immunohistochemical assessment, only 5 had a B-raf mutation whilst 10 had a K-ras mutation (Table 4). There was no difference in the distribution of K-ras mutations among the cases that showed nuclear and/or cytoplasmic pERK expression. Furthermore, all cases displayng cytoplasmic (11 cases) or both cytoplasmic and nuclear (8 cases) pERK expression had a wild-type B-raf. However, the presence of pERK immunoexpression, either nuclear or cytoplasmic was not correlated with the presence of either B-raf or K-ras mutations (Mann Whitney U test and Fisher's exact test, p > 0.10).

In the subset of the 63 patients for whom hMLH1 expression was available, 17 had a K-ras (12 of which preserved hMLH1 expression) and 7 a B-raf mutation (4 of which preserved hMLH1 expression) (Table 4). Accordingly, 64.71% of the cases (11/17) with K-ras mutation and 37.5% of the cases (3/8) with B-raf mutation preserved hMSH2 immunoreactivity (Table 4). However, the presence of hMLH1 or hMSH2 immunoexpression, was not correlated with the presence of B-raf and/or K-ras mutations (p > 0.10). Finally, the presence of K-ras *and/or *B-raf mutations could not be related to grade and stage (p > 0.10).

## Discussion

Various molecular markers have been proposed for the classification of colon cancer with regard to clinical course and outcome, including cell cycle as well as apoptotic regulators (c-FLIP, Ki67, COX-2) [22,23]. Deregulation of the MAPK signalling pathway has often been associated with oncogenic transformation [24]. In this regard, there is accumulating evidence involving ERK activation in the tumorigenesis of various human cancers such as prostate, breast, colon and ovary [25-29]. Furthermore, activating B-raf mutations are frequently detected in different tumor types such as melanomas, thyroid and colon carcinomas implying their significance as potential targets for anticancer treatment [30,31]. In the present study we used immunohistochemistry to examine the expression of total and activated ERK1/2 in a panel of 94 colorectal carcinomas in parallel with the expression of two MMR proteins (hMLh1 and hMSH2), as well as K-ras and B-raf mutations, which may lead to constitutive activation of MAPK pathway.

To the best of our knowledge, this appears to be the first study examining ERK expression in mismatch repair deficient and mismatch repair proficient colorectal cancer simultaneously with upstream gene alterations such as K-ras and B-raf mutations, which contribute to MAPK activation. In our cohort, ERK immunoreactivity was observed in the cytoplasm as well as in the nucleus in the majority of the cases (93% and 91% respectively). More than 78% of the examined cases displayed concurrent ERK nuclear and cytoplasmic immunoreactivity, in concordance with previous observations [29,30]. Increased levels of nuclear ERK positivity correlated with strong cytoplasmic ERK expression. The observed correlation between nuclear and cytoplasmic expression as well as the concurrent nuclear and cytoplasmic immunolocalization of ERK in the present series is compatible with its multiple functions targeting proteins localized in the cytoplasm as well as the nucleus of the cell. Moreover, pERK immunoreactivity was predominantly nuclear (68%) whereas cytoplasmic positivity was found in a subset of tumors (24%). This staining pattern is in line with previous investigations in colon as well as other neoplasms, namely, endometrial, head and neck tumors and melanomas [27,29,32,33]. Additionally, total nuclear or cytoplasmic ERK staining was independent of nuclear or cytoplasmic pERK status, in our cohort. This observation is in agreement with previous studies in non small cell lung and endometrial cancer as well as in melanomas and could been explained by the hypothesis that pERK immunopositivity could arise due to ERK hyperactivation rather than overexpression [29,30,32].

Furthermore we show that high nuclear, but not cytoplasmic, ERK immunopositivity is correlated with tumor stage (Table 1). Interestingly, our results demonstrate that nuclear pERK expression parallels tumor grade and tumor stage (Table 2, Figure 2) in keeping with findings of previous studies in NSCLC and prostate cancer [30,34]. This observation is in contrast with a previous investigation [25] in which pERK expression was not correlated with tumor stage, whereas there are other studies in various other tumors (breast, pancreatic, endometrial and ovarian cancer) that have failed to substantiate a positive correlation between pERK expression and classical clinicopathological parameters [26,32,35-37]. Taking into account that in our study normal colorectal mucosa displays minimal nuclear pERK expression, our results speak in favor of the potential role of this molecule in tumor evolution as well as, in the acquisition of a more aggressive phenotype in colorectal carcinogenesis. Along this line, it has been suggested that constitutively active ERKs are capable of affecting gene expression, being able to influence many of the hallmarks of carcinogenesis [35]. It could be hypothesised that as colorectal cancer progresses to a more advanced disease, an increase in the activation of the MAP kinase signal transduction pathway occurs [34]. Unfortunately, the statistical power of the present investigation is reduced due to the small cohort of pERK positive cases. Further studies of larger cohorts are warranted to confirm our results. Along this line, pERK expression has been shown to be associated with poor prognosis in colorectal carcinomas [27]. It has been speculated that the activation of the MAPK pathway initiates cellular processes, which could result in either favorable or worse clinical outcome, a fact due to the complex signal transduction network ERKs are involved in. In fact, adverse effects can be observed, depending on the intensity and duration of the promitogenic signal [10].

The aforementioned findings are quite significant in view of the fact that protein kinases represent a group of molecular targets characterized by a cancer specific potential, allowing the development of new generation chemotherapeutic agents acting as kinase inhibitors. The first oral multi-kinase inhibitor that targets Raf kinases have already been approved for the treatment of renal cell cancer, whereas these factors display a broad spectrum antitumor activity in colon, breast and non-small-cell lung cancer in xenograft models and also hepatocellular carcinoma and sarcoma [38,39]. In this context, the presence of B-raf mutations has been suggested as a possible surrogate marker of sensitivity to those drugs which target the ERK pathway at the level of Raf kinase [38,39].

The frequency of K-ras mutations detected in this study (about 23% of the examined cases) was comparable to that found in previous reports (Table 4) [39-41]. Furthermore, the presence of B-raf mutations in colorectal cancer is estimated to be about 10% of unselected colorectal cancers [42-44]. In particular, B-raf T1799A mutation (V600E) has been reported in 4% of microsatellite stable (MSS) tumors whereas in microsatellite unstable tumors the percentage rises up to 27-52% [42,44-46]. By analogy to those investigations we detected V600E B-raf mutations in about 7% of MSS tumors and in 21% of MSI unstable tumors. Interestingly, our cases exhibited only either B-raf or K-ras mutations in accordance with previous observations suggesting that these are mutually exclusive defects that probably exert equivalent effects in tumorigenesis [39,46].

Moreover, in our series the expression levels of total and activated ERK1/2 were independent of the mutation status of B-raf and K-ras genes. These results are in favor of the view that constitutive pERK activation occurs in a K-ras or B-raf -independent manner in a large subset of primary colon cancer cases. Recently, several negative regulators of the MAPK signalling pathway upstream of ERK at the level of Raf were identified, including Sprouty and Spred. Activation of these negative regulators inhibits phosphorylation of ERK1/2, even in the presence of mutation in K-ras gene [32,47]. This finding has also previously been observed in ulcerative colitis-related carcinomas [48]. The genetic nature of constitutive activation of the RAS/RAF/MEK/ERK pathway in colorectal tumors with no B-raf or K-ras mutation remains unknown, although it may in part due to increased activity of growth factor receptor induced cell proliferation pathways. It could be speculated that in cancer constitutive activation of MAP kinase could be triggered by upstream oncogenic regulators due to the presence of paracrine/autocrine growth factor stimulation, rather than Ras or B-raf mutations or components of the various other signal transduction pathways that interact with MAPK, since the mutation of K-ras and B-raf obviously constitutes one of multiple ways to activate this pathway.

In sporadic colorectal carcinogenesis B-raf mutations like K-ras mutations appear to occur early at the transition from small to medium size adenoma and are extremely frequent in so-called serrated adenomas [49]. According to the MSI colorectal pathway, MSI in sporadic tumors has been suggested to be mostly due to hypermethylation of the promoters of MMR genes and is correlated with B-raf mutations (44). However, in our study B-raf mutations were not correlated with loss of hMLH1 or hMSH2 protein, suggesting that the B-raf mutated cases of our cohort may belong to more than one colorectal carcinogenesis pathways.

## Conclusions

In this study, we have shown that mutations of the B-raf gene are not associated with mismatch-repair deficiency through loss of hMLH1 or hMSH2 expression. Disruption of the MAP kinase pathway-either through K-ras or B-raf mutation-was detected in 37% of all the examined cases, although the overexpression of total and activated ERK1/2 was not correlated with the mutational status of K-ras or B-Rraf genes. Interestingly, we present evidence that the expression of activated ERK expression parallels histological grade and stage in colon carcinomas, thus being implicated in tumor invasiveness as well as in the acquisition of a more aggressive phenotype. Our findings encourage prospective investigations to further elucidate and validate the potential role of pERK as a prognostic factor and therapeutic target in colon carcinogenesis.

## Competing interests

The authors declare that they have no competing interests.

## Authors' contributions

The manuscript was edited by GL, AAS Immunohistochemical staining was performed by PP and evaluated by GL, PK and NK. Molecular analysis was organized by AAS and performed by FG, MK and AS. Genomic DNA isolation was performed by MK. Statistical analysis was done by GL (MSc in Biostatistics). Clinical data of the patients were collected by NVM. The respective literature was collected by GA. The supervision and organization of the research program was performed by EP and PK. All the authors have read and approved the final manuscript.

## Supplementary Material

Additional file 1 **Supplementary table presenting all samples with their clinical information and the staining results**. This a comprehensive table in which all samples of the present study are listed along with their clinical information (i.e. age, gender) and the staining results of the examined proteins. Click here for file
