# Supplementary material for: ERK/pERK expression and B-raf mutations in colon adenocarcinomas: correlation with clinicopathological characteristics
Source: World J Surg Oncol. 2012 Feb 29;10:47. doi: 10.1186/1477-7819-10-47 (PMC3320554; doi:10.1186/1477-7819-10-47)
Supplement: Additional file 1 — Supplementary table presenting all samples with their clinical information and the staining results. This a comprehensive table in which all samples of the present study are listed along with their clinical information (i.e. age, gender) and the staining results of the examined proteins. [file 1477-7819-10-47-S1.DOC]

| **id** | **Grade** | **TNM** | **sex** | **age** | **location** | **kras** | **brafex15** | **brafex11** | **ERK cytoplasmic expression (%)** | **ERK nuclear expression (%)** | **pERK nuclear expression (%)** | **pERK cytoplasmic epxression (%)** | **hMLH1** | **hMSH2** |
| --- | --- | --- | --- | --- | --- | --- | --- | --- | --- | --- | --- | --- | --- | --- |
| 114018 | II/III | II | Γ | 88 | L | 0 | 0 | 0 | NA | NA | 1 | 0 | NA | NA |
| 114850 | II/III | IV | Γ | 60 | L | 1 | 0 | 0 | NA | NA | NA | NA | NA | NA |
| 178564 | II/III | II | Γ | 70 | L | 0 | 1 | 0 | NA | NA | NA | NA | 1 | 0 |
| 116178 | II/III | II | Α | 78 | L | 0 | 0 | 0 | NA | NA | 10 | 10 | NA | NA |
| 60712 | I | II | Α | 60 | L | 0 | 0 | 0 | 79 | 4 | NA | NA | 1 | NA |
| 107080 | I | I | Α | 81 | L | 0 | 1 | 0 | 55 | 70 | 0 | 0 | 1 | 1 |
| 78146 | II/III | III | Γ | 60 | L | 0 | 0 | 0 | 10 | 15 | 5 | 0 | 1 | NA |
| NA | II/III | III | Α | 60 | L | 0 | 0 | 0 | NA | NA | NA | NA | 1 | NA |
| 62917 | II/III | II | Γ | 60 | L | 1 | 0 | 0 | 0 | 3 | NA | NA | 1 | NA |
| 72411 | II/III | III | Α | 70 | L | 1 | 0 | 0 | 78 | 0 | NA | NA | 0 | 0 |
| 58264 | II/III | II | Α | 60 | L | 0 | 0 | 0 | 24 | 0 | 8 | 0 | 1 | NA |
| 78304 | II/III | III | Γ | 57 | L | 0 | 0 | 0 | 5 | 5 | NA | NA | 1 | 1 |
| 64752 | II/III | III | Α | 60 | L | 0 | 0 | 0 | 61 | 16 | NA | NA | NA | NA |
| 75934 | II/III | III | Γ | 57 | L | 0 | 0 | 0 | 95 | 40 | 5 | 0 | 0 | 1 |
| 3831 | II/III | III | Α | 60 | L | 1 | 0 | 0 | NA | NA | 5 | 1 | 0 | 0 |
| 72899 | II/III | III | Α | 76 | L | 1 | 0 | 0 | 10 | 0 | NA | NA | 1 | 1 |
| 60330 | I | II | Α | 60 | L | 1 | 0 | 0 | 91 | 80 | NA | NA | 1 | NA |
| 106943 | II/III | II | Γ | 75 | L | 0 | 0 | 0 | NA | NA | 1 | 0 | 1 | 1 |
| 72988 | II/III | II | Γ | 70 | L | 0 | 0 | 0 | 58 | 20 | NA | NA | NA | NA |
| 385 | II/III | II | Α | 58 | L | 0 | 0 | 0 | NA | NA | NA | NA | 0 | 0 |
| 74345 | II/III | IV | Γ | 63 | L | 0 | 0 | 0 | 70 | 15 | NA | NA | 0 | 1 |
| 66003 | II/III | III | Α | 60 | L | 1 | 0 | 0 | 90 | 65 | NA | NA | 0 | 1 |
| 107883 | II/III | III | Α | 60 | L | 0 | 0 | 0 | 87 | 15 | NA | NA | NA | NA |
| 107210 | II/III | I | Α | 59 | L | 0 | 0 | 0 | 0 | 47 | 10 | 0 | 1 | 1 |
| 53889 | II/III | III | Γ | 60 | L | 0 | 0 | 0 | 51 | 18 | 1 | 0 | 1 | 1 |
| 64073 | II/III | II | Γ | 60 | L | 0 | 0 | 0 | 80 | 70 | 0 | 15 | 1 | NA |
| 115543 | I | II | Α | 74 | L | 0 | 0 | 0 | 70 | 70 | NA | NA | 1 | 1 |
| 107873 | II/III | I | Α | 60 | L | 0 | 0 | 0 | 75 | 32 | 0 | 0 | 0 | 0 |
| 77264 | II/III | III | Α | 63 | L | 0 | 0 | 0 | 81 | 1 | NA | NA | 1 | 1 |
| NA | II/III | II | Α | 60 | L | 0 | 0 | 0 | NA | NA | NA | NA | NA | NA |
| 49540 | II/III | III | Α | 60 | L | 1 | 0 | 0 | 90 | 90 | NA | NA | 1 | 1 |
| 60347 | II/III | III | Γ | 60 | L | 0 | 0 | 0 | 78 | 85 | 5 | 5 | 1 | 1 |
| 111866 | II/III | II | Γ | 60 | L | 0 | 1 | 0 | 0 | 5 | NA | NA | 1 | 0 |
| 111814 | I | II | Α | 60 | L | 1 | 0 | 0 | 0 | 5 | NA | NA | 1 | 0 |
| NA | II/III | III | Α | 60 | R | 0 | 0 | 0 | NA | NA | NA | NA | 1 | NA |
| 107810 | II/III | II | Γ | 83 | R | 0 | 0 | 0 | 88 | 18 | 10 | 10 | 1 | 0 |
| 106991 | II/III | III | Γ | 56 | R | 1 | 0 | 0 | 51 | 1 | 5 | 0 | 1 | 1 |
| 114015 | II/III | II | Γ | 60 | R | 0 | 0 | 0 | NA | NA | NA | NA | NA | NA |
| 177128 | II/III | III | Γ | 53 | R | 0 | 1 | 0 | NA | NA | NA | NA | 0 | 1 |
| 74627 | II/III | III | Γ | 56 | R | 0 | 0 | 0 | 45 | 50 | 1 | 0 | 0 | 0 |
| 116593 | I | III | Α | 60 | R | 0 | 0 | 0 | NA | NA | NA | NA | NA | NA |
| 54892 | II/III | II | Α | 60 | R | 0 | 1 | 0 | 10 | 5 | 5 | 0 | 1 | NA |
| 73570 | II/III | III | Α | 60 | R | 0 | 0 | 0 | 62 | 40 | 0 | 5 | 0 | 1 |
| 75523 | II/III | III | Α | 44 | R | 1 | 0 | 0 | 90 | 60 | 1 | 0 | 1 | 1 |
| 61831 | II/III | III | Α | 60 | R | 1 | 0 | 0 | NA | NA | 10 | 0 | 1 | 0 |
| 63589 | II/III | III | Α | 60 | R | 0 | 0 | 0 | 70 | 50 | 0 | 0 | 1 | 0 |
| NA | II/III | III | Γ | 60 | R | 0 | 0 | 0 | NA | NA | NA | NA | 1 | NA |
| 107955 | II/III | II | Α | 57 | R | 0 | 0 | 0 | 88 | 0 | 0 | 0 | 1 | 1 |
| 114813 | II/III | III | Γ | 61 | R | 0 | 0 | 0 | NA | NA | NA | NA | NA | NA |
| 179300 | I | I | Γ | 45 | R | 0 | 1 | 0 | NA | NA | NA | NA | NA | 0 |
| 107882 | II/III | III | Γ | 70 | R | 0 | 0 | 0 | NA | NA | NA | NA | NA | NA |
| 67442 | II/III | III | Γ | 60 | R | 0 | 0 | 0 | 82 | 58 | 20 | 0 | 1 | NA |
| 133068 | II/III | III | Γ | 57 | R | 1 | 1 | 0 | NA | NA | NA | NA | 0 | 1 |
| 106855 | II/III | II | Α | 77 | R | 1 | 0 | 0 | 79 | 7 | 1 | 0 | NA | 1 |
| 111754 | II/III | II | Γ | 60 | R | 0 | 0 | 0 | 0 | 8 | 0 | 0 | 1 | 1 |
| 107006 | II/III | II | Α | 73 | R | 1 | 0 | 0 | 62 | 7 | 0 | 10 | 0 | 1 |
| 74519 | II/III | III | Γ | 60 | R | 0 | 0 | 0 | 65 | 1 | 10 | 10 | 1 | 1 |
| 61527 | I | II | Γ | 60 | R | 0 | 0 | 0 | 53 | 29 | 0 | 0 | 0 | NA |
| 179068 | II/III | II | Γ | 64 | R | 0 | 1 | 0 | NA | NA | NA | NA | NA | NA |
| 109261 | II/III | I | Α | 60 | R | 0 | 0 | 0 | NA | NA | NA | NA | NA | NA |
| 53414 | II/III | IV | Α | 60 | R | 0 | 0 | 0 | 60 | 4 | NA | NA | 0 | NA |
| 111885 | II/III | I | Α | 61 | R | 0 | 0 | 0 | 40 | 40 | 0 | 0 | 1 | 1 |
| NA | II/III | II | Γ | 60 | R | 0 | 0 | 0 | NA | NA | NA | NA | NA | NA |
| 111868 | II/III | III | Α | 47 | R | 0 | 0 | 0 | NA | NA | 1 | 0 | NA | NA |
| 106694 | II/III | III | Γ | 60 | R | 0 | 0 | 0 | NA | NA | NA | NA | NA | NA |
| NA | II/III | III | Γ | 60 | R | 0 | 0 | 0 | NA | NA | NA | NA | 0 | NA |
| 175230 | II/III | II | Α | 41 | R | 0 | 1 | 0 | NA | NA | 5 | 0 | NA | NA |
| 114797 | II/III | III | Α | 48 | R | 0 | 0 | 0 | NA | NA | NA | NA | NA | NA |
| 60539 | II/III | III | Α | 60 | R | 1 | 0 | 0 | 67 | 44 | 0 | 0 | 1 | 1 |
| 70834 | II/III | IV | Α | 60 | R | 0 | 0 | 0 | 65 | 5 | NA | NA | NA | 0 |
| 4122 | II/III | II | Α | 35 | R | 0 | 0 | 0 | 70 | 70 | 15 | 0 | NA | NA |
| 62582 | II/III | II | Α | 60 | R | 0 | 0 | 0 | 96 | 2 | NA | NA | 1 | NA |
| 111784 | I | I | Α | 60 | R | 1 | 0 | 0 | NA | NA | 0 | 0 | 1 | 1 |
| 114870 | II/III | II | Α | 60 | R | 1 | 0 | 0 | NA | NA | NA | NA | NA | NA |
| 106985 | II/III | II | Α | 60 | R | 0 | 1 | 0 | 71 | 2 | 1 | 0 | 0 | 0 |
| 78403 | II/III | III | Α | 60 | R | 0 | 0 | 0 | 75 | 90 | 15 | 0 | 1 | 1 |
| 55766 | II/III | III | Α | 60 | R | 0 | 0 | 0 | 40 | 0 | NA | NA | 1 | 1 |
| 76895 | II/III | III | Α | 60 | R | 0 | 0 | 0 | NA | NA | NA | NA | NA | 1 |
| 42432 | II/III | II | Γ | 60 | R | 0 | 0 | 0 | NA | NA | 1 | 0 | 1 | 1 |
| 78537 | II/III | III | Α | 51 | R | 0 | 0 | 0 | 50 | 5 | 2 | 2 | NA | NA |
| 72747 | II/III | II | Γ | 60 | R | 0 | 0 | 0 | 17 | 60 | NA | NA | 1 | 0 |
| 114855 | II/III | II | Α | 60 | R | 1 | 0 | 0 | NA | NA | NA | NA | NA | NA |
| NA | II/III | III | Α | 60 | R | 0 | 0 | 0 | NA | NA | NA | NA | NA | NA |
| 75427 | II/III | III | Α | 60 | R | 1 | 0 | 0 | 65 | 55 | 1 | 10 | 1 | 1 |
| 142769 | II/III | III | Α | 47 | R | 1 | 1 | 0 | NA | NA | NA | NA | NA | 0 |
| 57238 | II/III | III | Γ | 60 | R | 1 | 0 | 0 | 36 | 0 | 2 | 0 | 1 | 0 |
| 127559 | II/III | II | Γ | 58 | R | 0 | 1 | 0 | NA | NA | 3 | 0 | NA | NA |
| 107794 | I | III | Γ | 76 | R | 0 | 0 | 0 | 97 | 6 | 0 | 0 | 1 | 0 |
| 78208 | II/III | III | Α | 60 | R | 0 | 0 | 0 | NA | NA | NA | NA | 0 | 1 |
| 80303 | II/III | II | Γ | 60 | R | 0 | 0 | 0 | 90 | 0 | 5 | 5 | 0 | 1 |
| 109072 | II/III | III | Γ | 40 | R | 0 | 0 | 0 | NA | NA | 0 | 0 | 1 | 1 |
| 57724 | II/III | III | Α | 60 | R | 0 | 0 | 0 | NA | NA | NA | NA | NA | NA |
| 108341 | II/III | II | Γ | 60 | R | 0 | 0 | 0 | NA | NA | NA | NA | NA | NA |
| 66227 | II/III | III | Γ | 60 | R | 0 | 0 | 0 | 75 | 7 | 1 | 0 | 1 | 1 |
